# Tossing the coin of extended-spectrum β-lactamase: prevalence of extended-spectrum β-lactamase-producing Klebsiella pneumoniae isolated from patients with sepsis

**DOI:** 10.1099/acmi.0.000962.v3

**Published:** 2025-02-19

**Authors:** Beatrice Achan, Tonny Luggya, Robert Innocent Ebwongu, Simon Sekyanzi, Henry Kajumbula

**Affiliations:** 1 Department of Medical Microbiology, School of Biomedical Sciences, College of Health Sciences, Makerere University Kampala, Kampala, Uganda

**Keywords:** *Klebsiella pneumoniae*, AMR, extended-spectrum *β*-lactamase

## Abstract

**Background.***Klebsiella pneumoniae* is part of the ESKAPE (*Enterococcus faecium*, *Staphylococcus aureus*, *K. pneumoniae*, *Acinetobacter baumannii*, *Pseudomonas aeruginosa* and *Enterobacter* spp.) group of multidrug-resistant (MDR) pathogens. *K. pneumoniae* is the leading cause of antimicrobial resistance-associated mortality and the second leading cause of nosocomial bloodstream infections (BSIs), globally and in sub-Saharan Africa. Therefore, it was aimed to determine the antibiotic resistance patterns of *K. pneumoniae* isolated from blood cultures of patients with features of sepsis at Mulago National Referral Hospital, Uganda.

**Methods.** The cross-sectional study on patients with features of sepsis utilized *K. pneumoniae* (*n*=30) isolated from positive blood culture specimens. The antibiotic resistance profile was determined by the Clinical and Laboratory Standards Institute’s Kirby–Bauer disc diffusion method, which was used to classify the isolates as susceptible, intermediate and resistant. *K. pneumoniae* isolates that were resistant to third-generation cephalosporins were subjected to extended-spectrum *β*-lactamase (ESBL) screening and confirmation using the double-disc synergy test using cefotaxime, ceftazidime, ceftriaxone, cefotaxime–clavulanic acid and ceftazidime–clavulanic acid. The results were analysed for frequencies.

**Results.***K. pneumoniae* isolates showed emerging resistance to imipenem at 13% (4 out of 30) followed by amikacin at 17% (5 out of 30). There was intermediate resistance to gentamycin at 60% (18 out of 30). However, *K. pneumoniae* showed the highest resistance to piperacillin at 100% (30 out of 30) followed by sulphamethoxazole-trimethoprim and cefepime, both showing a percentage of 97% (29 out of 30). Up to 16 out of 30 (53.3%) of *K. pneumoniae* were positive for ESBL production, whilst 14 out of 30 (46.7%) were negative.

**Conclusion.** There was a high prevalence of antibiotic-resistant *ESBL*-producing *K. pneumoniae* isolates from BSI of patients with features of sepsis in Uganda’s Mulago National Referral Hospital.

## Data Summary

All data generated or analysed during this study are included in this published article and its supplementary information file.

## Introduction

*Klebsiella pneumoniae* is part of the ESKAPE (*Enterococcus faecium*, *Staphylococcus aureus*, *K. pneumoniae*, *Acinetobacter baumannii*, *Pseudomonas aeruginosa* and *Enterobacter* spp.) group of multidrug-resistant (MDR) pathogens [1], which are frequent aetiologies of bloodstream infections (BSIs), which are second to lower respiratory tract infections in hospitalized patients [2]. *K. pneumoniae* is the leading cause of antibiotic resistance-associated mortality, ranked as the second leading cause of Gram-negative bacteraemia and, overall, the third commonest cause (after *Escherichia coli*) of bacteraemia [3]. *K. pneumoniae* BSIs are usually secondary to focal infections from other anatomical sites including the urinary, respiratory, skin and/or gastrointestinal tract infections in patients whose immune systems are compromised. Alternatively, sources of *K. pneumoniae* BSIs are idiopathic [4,5].

Over the past two decades, the importance of *K. pneumoniae* as a BSI pathogen seems to be rising due to its association with high mortality rate and antimicrobial resistance [6]. There are also several reports, including the World Health Organization’s Global Antimicrobial Resistance Surveillance System reports, that recent years have witnessed increasing rates of antibiotic-resistant *K. pneumoniae*, significantly, the strains producing extended-spectrum β-lactamases (ESBL) [7]. ESBLs are plasmid-mediated enzymes that confer resistance to all penicillins, cephalosporins, sulbactams, clavulanic acid combinations and monobactams, for example, aztreonam [8].

Globally, the burden of *ESBL*-producing *K. pneumoniae* (*ESBL-Kp*) ranges from 4.9% in Canada, 7.6% in the USA and 22% in Europe to 45.4% in Latin America [9]. However, in Africa, the prevalence of *ESBL* is not clear because of the paucity of data. A study in Nigeria reported a 50% prevalence of *ESBL-Kp* [10], whilst in Ethiopia, the finding showed 17.1% [11]. In Ivory Coast, there was a high prevalence of *ESBL-Kp* up to 84% [12]. Like Ivory Coast, in Uganda, a previous study in 2015 reported a high prevalence of *ESBL-Kp* at 72.7% [13] and recently in 2023, at 50% [14]. Resistance to third-generation cephalosporins is particularly important because of AMR-associated mortality. Therefore, we aimed to determine the prevalence of *ESBL-Kp* clinical isolates from patients with features of sepsis at a National Referral and Teaching Mulago Hospital in Uganda.

## Methods

### Study design

Between September 2020 and September 2021 inclusive, patients with features of sepsis – temperature >38 °C or <36 °C, tachycardia (heart rate >90 beats/min), tachypnoea (respiratory rate) >20 breaths/min/PaCO_2_ <32 mm Hg, WBC >12 000 cells/mm^3^ or <4000 cells/mm^3^ or <10 % immature (band) forms and suspected focus of infection [15] – were enrolled at the intensive care unit (ICU), infectious disease wards and the Uganda Cancer Institute, Mulago National Referral Hospital. The sample size for participants was estimated using the Kish Leslie formula for cross-sectional studies [16]. The calculation was based on an estimated prevalence of bacteremia amongst ICU and cancer patients of 15%, with a margin of error of 3% at 95% CIs. The sample size was therefore estimated to be 385 participants for patients with features of sepsis. However, as the study objective is focused on * K. pneumoniae* isolated within a year of the study, the sample size is not considered for this report.

From each patient, a total of 20 ml of blood was collected from two venepuncture sites (10 ml per site) after skin disinfection with 70% isopropyl alcohol followed by 2% tincture of iodine. The blood was aseptically added to BD BACTEC plus aerobic blood culture bottles and transported at room temperature within 2 h of collection for laboratory analysis.

### Laboratory methods

Procedures for blood culture, *K. pneumoniae* isolation, identification and antibiotic susceptibility testing were performed at the College of American Pathologists-accredited Clinical Microbiology Laboratory, Department of Medical Microbiology, College of Health Sciences, Makerere University, which is accredited by the College of American Pathologists.

### Blood culture in the automatic BD BACTEC system

Blood culture specimens, which were collected in the BD BACTEC™ Plus Aerobic/F, were loaded onto the BD BACTEC 9120 blood culture system immediately upon transportation to the laboratory. Then, blood culture bottles were incubated in the BD BACTEC 9120 blood culture system, which gives a signal upon laser detection of any growth, usually within 1–3 days. After 7 days of no laser signal, the blood culture bottles that were flagged negative by the BD BACTEC 9120 blood culture system were removed.

### Isolation and identification of *K. pneumoniae*

The positive blood culture specimens were Gram-stained (*K. pneumoniae* are Gram-negative rods), then sub-cultured on MacConkey agar and incubated at 37 °C for 18–24 h in ambient air. Pink lactose-fermenting colonies on MacConkey agar were identified using the following biochemical tests: positive citrate utilization test; yellow slant, red butt on triple sugar iron agar, no HS_2_ production and presence of gas bubbles on triple sugar iron agar, indole negative and lack of motility on sulphur indole motility smedium; and positive urea hydrolysis on urea agar as *K. pneumoniae* [17].

### Antibiotic resistance testing of *K. pneumoniae*

*K. pneumoniae* were tested for antibiotic resistance profiles on Mueller–Hinton agar (MHA) by the Kirby–Bauer disc diffusion technique as recommended by the Clinical Laboratory Standards Institute (CLSI) guideline and by following the Clinical Microbiology Laboratory standard operating procedures. Briefly, the Kirby–Bauer disc diffusion method involved the preparation of the 0.5 McFarland standard of *K. pneumoniae* by picking two to three colonies from pure cultures and emulsifying them in sterile normal saline. The suspension was streaked onto MHA to create a lawn; then, a sterile pair of forceps was used to pick and place on MHA, the different discs of antibiotics are as follows: cefuroxime (CXM), ciprofloxacin (CIP), imipenem (IMP), chloramphenicol (C), gentamicin (CN), cefepime (FEP), piperacillin (PRL), amikacin (AK), ceftazidime (CAZ), amoxicillin/clavulanic acid (AMC), ceftriaxone (CRO), tazobactam-piperacillin (TZP-PRL). The plates were incubated aerobically at 35–37 °C for 18–24 h. The zones of inhibition diameters around each disc were then measured using a ruler and compared against the zone diameter interpretative standards according to the laboratory’s CLSI-based Standard Operating Procedure documents as resistant (R), intermediate (I) and susceptible (S), and the results were presented in a bar graph.

### ESBL*-Kp* screening

The double-disc synergy test was used to determine the ESBL-producing *K. pneumoniae,* confirmed to be resistant to third-generation cephalosporins. TZP-PRL disc was placed on MHA plates inoculated with 0.5 McFarland *K. pneumoniae* and CAZ, CRO and cefotaxime (CTX) placed at 1.5 cm. The *K. pneumoniae* isolate was identified as positive for ESBL production if there was clearance between the cephalosporins and TZP-PRL disc or negative if there was no clearance between the cephalosporins and TZP-PRL. The results of the proportions of the positive and negative ESBL-*Kp* were represented in a pie chart.

### Quality control

The identification and antibiotic susceptibility of *K. pneumoniae* were run alongside the ESBL-positive control strain, *K. pneumoniae* ATCC 700603, and the negative control strain, *E. coli* 25922.

### Data analysis

The data were entered into Microsoft Excel 365 (2019 version) and checked for completeness and correctness. The data were then analysed for mean, mode and other measures of central tendency.

### Ethical and consent

The study was approved by the ethics committees of the School of Biomedical Sciences, Makerere University College of Health Sciences (SBS 639) on 23 July 2020 and the Uganda National Council for Science and Technology (HS1127ES) on 24 February 2021. Patients gave written informed consent before specimen collection. Where the patients could not write, a thumbprint was used to gain informed consent. Laboratory identification numbers were used to accompany patients’ blood culture specimens into the laboratory instead of patient names for purposes of confidentiality.

## Results

*K. pneumoniae* was isolated from 17 (56%) males and 13 (44%) females. The median and mean ages were 29 and 31 years old, respectively, with a range of 18–59 years. Most of the patients, 43% (13 out of 30), with *K. pneumoniae* BSI were between 28 and 37 years, whilst the least infections were observed in patients aged 48–57 years old, contributing to 3% (Table 1).

**Table 1.** Table showing patient characteristics and hospital units of the patients with features of sepsis from whom *K. pneumoniae* was isolated

*Patient characteristics*	*Description of the patient characteristics*		
***Age***	**Age group (years**)	**Frequency (*n*=30**)	**Percentage (%)**
	18–27	11	37
	28–37	13	43
	38–47	3	10
	48–57	1	3
	58–67	2	7
***Gender***			
**Male**		17	57
**Female**		13	43
***Hospital unit***			
**ICU**		15	50
**Uganda Cancer Institute**		9	30
**Infectious disease ward**		6	20

**Fig. 1.**
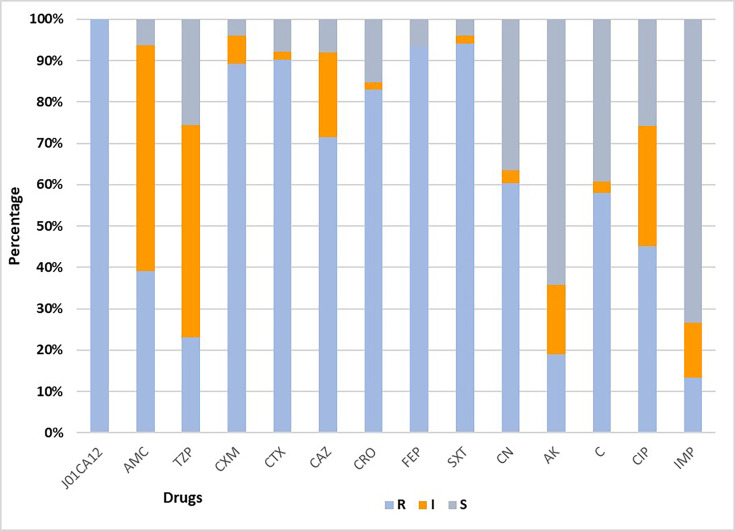
Antimicrobial susceptibility profile of *K. pneumoniae*. Antimicrobial susceptibility profiles of *K. pneumoniae* were tested by CLSI Kirby–Bauer disc diffusion and the zone diameters classified as R, I and S for each antibiotic. Names of the antibiotics: J01CA12-PRL, AMC, TZP-PRL, cefixime (CXM), CTX, CAZ, CRO, FEP, sulphamethoxazole-trimethoprim (SXT), CN, AK, C, CIP and IMP.

A total of 32 *K. pneumoniae* species were isolated from positive blood culture specimens. Of the 32 *K. pneumoniae* isolates, demographic data did not match for one isolate, whilst one isolate failed to grow when purity plated. Collectively, there was a total of two invalid results, leaving 30 (*n*=30) *K. pneumoniae* isolates that were tested for antibiotic susceptibility profiles using the CLSI-based Kirby–Bauer disc diffusion guideline on MHA. The readings of antibiotic susceptibility profiles of *K. pneumoniae* were interpreted as S, I or R as shown in Fig. 1.

*K. pneumoniae* showed the highest resistance to PRL (100%) followed by SXT and FEP both showing a percentage of 94%. However, resistance to carbapenems, IMP (13%), has emerged as shown in Fig. 1. The *K. pneumoniae* isolates from diagnostic specimens expressed the highest resistance to the lowest class generation drugs in penicillins and cephalosporins. The increasing trend of resistance to *β*-lactam antibiotics is worrying because *β*-lactam resistance is considered difficult to treat. Furthermore, there is emerging resistance to carbapenems, which is of concern.

**Fig. 2.**
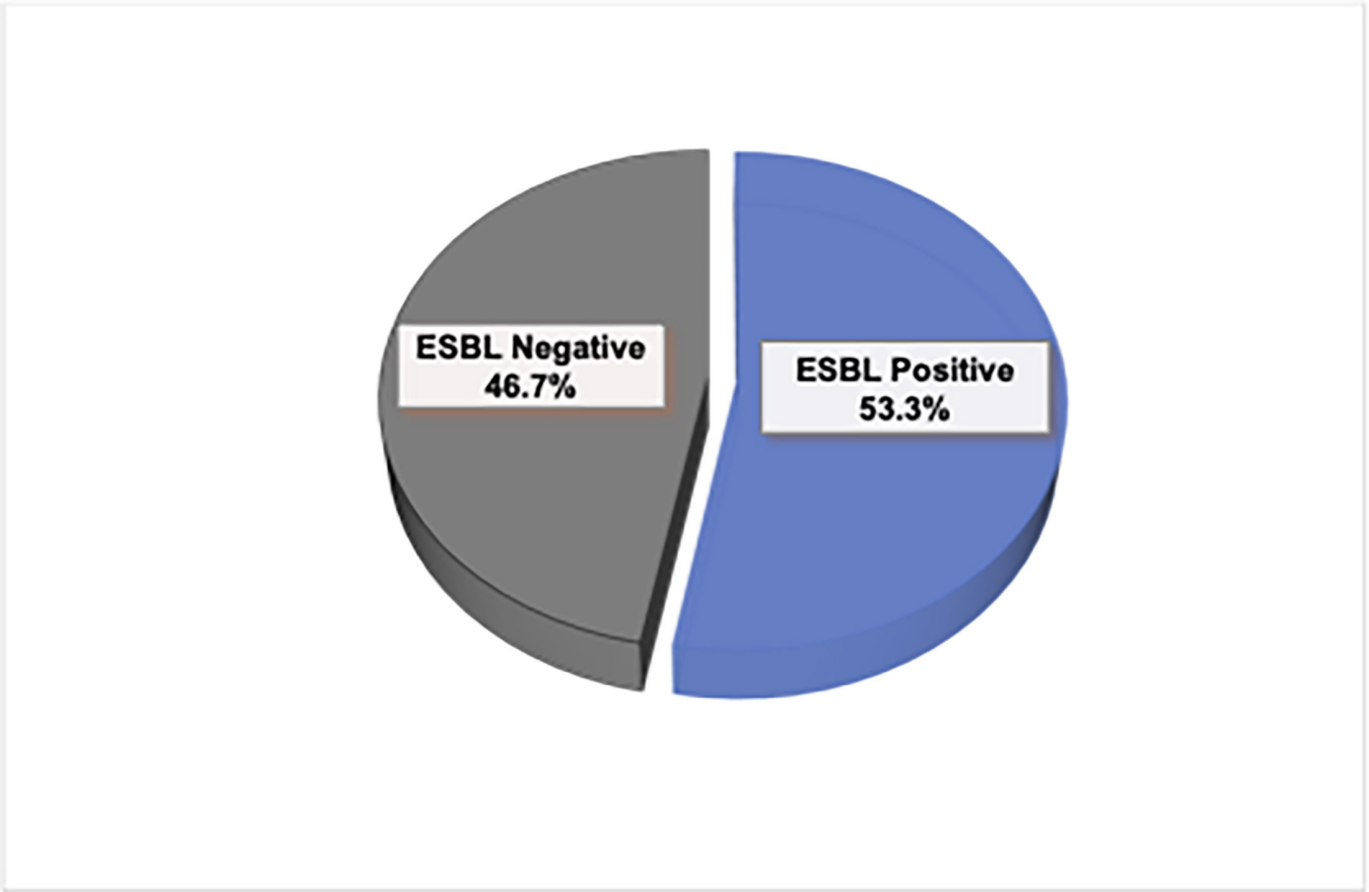
Proportion of *ESBL*-producing *K. pneumoniae*. *K. pneumoniae* resistant to third-generation cephalosporins were screened for ESBL production using the double-disc synergy test, and the results are presented as % as shown in the pie chart.

The *K. pneumoniae* isolates that were resistant to third-generation cephalosporins were subjected to ESBL screening and confirmation by using the double-disc synergy test. Out of 30, 16 (53.3%) were positive for ESBL production, whilst 14 out of 30 (46.7%) were negative (Fig. 2).

## Discussion

The risk of *K. pneumoniae* BSI was slightly higher in male patients at 57% (17 out of 30) than in their female counterparts. Our finding is consistent with previous studies that have reported gender differences with men being at a higher risk for BSI, associated with detection bias [15]. Urinary tract infections (UTIs), which are the primary sources of BSIs, are less detected in men because UTI is thought to be less common in them [15]. The undiagnosed and untreated UTI could act as a focus of infection BSI, hence the higher rates in males. Furthermore, in our study, the higher male number with BSI may also be reflective of the higher patient population, with 50% (15 out of 30) admitted to the ICU, where most of the study participants were recruited from. Most patients admitted to the ICU were due to injuries sustained in road traffic accidents, and previous studies have shown that more males are involved in motor accidents in Uganda [16].

However, in contrast with prior investigations, we found that advancing age was not a risk factor for *K. pneumoniae* bacteraemia as demonstrated by the prevalent age category being 28–37-year-old patients. This finding was due to the skewed patient population composed predominantly of young males (57%), who were admitted to the ICU during this study period (Table 1).

Resistance of *K. pneumoniae* to PRL, SXT and FEP is due to these drugs being accessible over the counter for empirical treatment of infections of the bloodstream and other sites, their inexpensive cost and broad-spectrum activities [17]. The resistance of * K. pneumoniae* to PRL is mediated through the production of *β*-lactamase enzyme [18], evidenced by the decreased resistance of *K. pneumoniae* from 100% to 23% when a *β*-lactam/*β*-lactamase inhibitor combination of TZP-PRL is used (Fig. 1).

Our finding of the high prevalence of ESBL amongst the isolates of *K. pneumoniae* means that the resistant strains were possibly transmissible because the *ESBL* gene is coded on a plasmid. A plasmid is a mobile genetic element that promotes gene exchange (in a setting of compromised infection control practices, which can occur amongst hospitalized patients in a crowded setting) [14]. Our finding on the high prevalence of *ESBL*-producing *K. pneumoniae* is consistent with a previous study in Jordanian hospitals that reported a high prevalence of ESBL-*Kp* up to 70% [19], whilst in Latin America, the prevalence was 45% [9]. However, when compared with developed countries such as the USA and Canada, our finding on the prevalence of *ESBL-Kp* at 30% was higher than in Europe (22.6%), the USA (7.6%) and Canada (4.9%) [9]. In Africa, the prevalence of *ESBL* is not clear because of the paucity of data; however, a study in Nigeria reported a 50% prevalence [10]. The findings of our study agree with a previous study in which it was observed that *K. pneumonia* has the highest prevalence of ESBL amongst Enterobacteriaceae [10]. The differences in the high and low prevalence of *ESBL-Kp* in Uganda, Nigeria, Latin America, the USA, Canada and Europe may be due to limited antibiotic stewardship. For example, in Uganda, antibiotics are accessible over the counter in pharmacies [17,20], which initiates high rates of carriage of resistance of normal flora, which are the ready sources of infections. Amongst hospitalized patients, there is limited capacity for infection control practices in Africa and Latin America compared to the USA, Canada and Europe that are developed, hence the higher rates of spread of resistance [21,22]. Our findings are also consistent with previous research from Uganda; one study reported that 90.91% of *K. pneumoniae* were resistant to PRL (90.91%), and ~84% of the isolates were resistant to CXM, CAZ and CTX [23]. Other studies reported that up to 50% of the isolates carried an *ESBL* gene responsible for spreading resistance attributable to limited antibiotic stewardship, infection prevention and control practices, both in colonization and disease [24,26].

## Conclusion

Our study shows a high antibiotic resistance profile, highlighted by >50% prevalence of ESBL-producing *K. pneumoniae* isolated from patients with features of sepsis at Mulago National Referral Hospital of Uganda. *K. pneumoniae* is part of the ESKAPE MDR pathogens; therefore, there is a need to perform antibiotic susceptibility testing for the guidance of antibiotic therapy and to continuously monitor resistance trend. A limitation of the study is that genotypic typing to attribute the resistance phenotypes to genes and to understand the relatedness of the *ESBL-Kp* strains at molecular levels to accurately inform infection control practices was not performed.

## supplementary material

10.1099/acmi.0.000962.v3 Uncited Supplementary Material 1.

## Abbreviations

AK: amikacin
AMC: amoxicillin/clavulanic acid
AMR: antimicrobial resistance
BSI: bloodstream infection
C: chloramphenicol
CAZ: ceftazidime
CIP: ciprofloxacin
CLSI: Clinical Laboratory Standards Institute
CN: gentamicin
CRO: ceftriaxone
CTX: cefotaxime
CXM: cefuroxime
ESBL: extended-spectrum *β-*lactamase
ESBL-*Kp*: extended-spectrum *β-*lactamase-producing *Klebsiella pneumoniae*
ESKAPE: *Enterococcus faecium*, *Staphylococcus aureus*, *Klebsiella pneumoniae*, *Acinetobacter baumannii*, *Pseudomonas aeruginosa* and *Enterobacter* spp.
FEP: cefepime
I: intermediate
ICU: intensive care unit
IMP: imipenem
MHA: Mueller–Hinton agar
PRL: piperacillin
R: resistant
S: susceptible
SXT: sulphamethoxazole-trimethoprim
TPZP-PRL: tazobactam-piperacillin
UTIs: urinary tract infections
