# Corrigendum: Tossing the coin of extended-spectrum β-lactamase: prevalence of extended-spectrum β-lactamase-producing Klebsiella pneumoniae isolated from patients with sepsis

**DOI:** 10.1099/acmi.0.001047

**Published:** 2025-05-07

**Authors:** Beatrice Achan, Tonny Luggya, Robert Innocent Ebwongu, Simon Sekyanzi, Henry Kajumbula

**Affiliations:** 1 Department of Medical Microbiology, School of Biomedical Sciences, College of Health Sciences, Makerere University Kampala, Kampala, Uganda

European and Developing Countries Clinical Trials Partnership, one of the funders of this research, has requested a change of the funding information provided in the article.

The updated funding information is:

This project is part of the EDCTP2 programme supported by the European Union (grant number TMA2018CDF-2371), NURTURE/NIH Fogarty International Centre training grant Number D43TW010132 and, Uganda Government’s Makerere Research and Innovation Fund.

Previously, the funding information was:

This project has received funding from the European Union’s Horizon 2020 research and innovation programme under grant agreement Number TMA2018CDF2371, NURTURE/NIH Fogarty International Centre training grant Number D43TW010132 and, Uganda Government’s Makerere Research and Innovation Fund.

The version of record was updated to reflect this correction.

